# OsNAC103, an NAC transcription factor negatively regulates plant height in rice

**DOI:** 10.1007/s00425-023-04309-7

**Published:** 2024-01-09

**Authors:** Yan Li, Liming Zhao, Chiming Guo, Ming Tang, Wenli Lian, Siyu Chen, Yuehan Pan, Xiaorong Xu, Chengke Luo, Yin Yi, Yuchao Cui, Liang Chen

**Affiliations:** 1 Xiamen Key Laboratory for Plant Genetics, School of Life Sciences, Xiamen University, Xiamen, 361102 China; 2 Fujian Key Laboratory of Subtropical Plant Physiology and Biochemistry, Fujian Institute of Subtropical Botany, Xiamen, 361006 China; 3 Key Laboratory of National Forestry and Grassland Administration On Biodiversity Conservation in Karst Mountainous Areas of Southwestern, School of Life Science, Guizhou Normal University, Guiyang, 550025 China; 4 Agricultural College, Ningxia University, Yinchuan, 750021 China

**Keywords:** Cell cycle, Cytokinins, Gibberellins, Phytohormones crosstalk, Plant development

## Abstract

**Main conclusion:**

*OsNAC103* negatively regulates rice plant height by influencing the cell cycle and crosstalk of phytohormones.

**Abstract:**

Plant height is an important characteristic of rice farming and is directly related to agricultural yield. Although there has been great progress in research on plant growth regulation, numerous genes remain to be elucidated. NAC transcription factors are widespread in plants and have a vital function in plant growth. Here, we observed that the overexpression of *OsNAC103* resulted in a dwarf phenotype, whereas RNA interference (RNAi) plants and *osnac103* mutants showed no significant difference. Further investigation revealed that the cell length did not change, indicating that the dwarfing of plants was caused by a decrease in cell number due to cell cycle arrest. The content of the bioactive cytokinin N^6^-Δ^2^-isopentenyladenine (iP) decreased as a result of the cytokinin synthesis gene being downregulated and the enhanced degradation of cytokinin oxidase. *OsNAC103* overexpression also inhibited cell cycle progression and regulated the activity of the cell cyclin *OsCYCP2;1* to arrest the cell cycle. We propose that *OsNAC103* may further influence rice development and gibberellin–cytokinin crosstalk by regulating the *Oryza sativa homeobox 71* (*OSH71*). Collectively, these results offer novel perspectives on the role of *OsNAC103* in controlling plant architecture.

**Supplementary Information:**

The online version contains supplementary material available at 10.1007/s00425-023-04309-7.

## Introduction

The plant height of rice plants is regulated by various factors. In addition to environmental conditions, numerous genes regulate plant height, and most of the regulatory pathways involve phytohormones, such as gibberellins, cytokinins, brassinosteroids, and auxins, which affect stem elongation and thickness (Margis-Pinheiro et al. 2005; Tanabe et al. 2005; Peng et al. 2014; Wang et al. 2018). Cytokinins influence plant height, development, and senescence, and have a crucial function in the cell cycle (Hwang et al. 2012; Liu et al. 2023). Cytokinins exist as free bases, ribosides, and ribotides (Miyawaki et al. 2006). Free-base cytokinins represent the biologically active forms, such as N^6^-Δ^2^-isopentenyladenine (iP), *cis*-zeation (*c*Z), and *trans*-zeation (*t*Z). However, ribosides, such as isopentenyladenine riboside (iPR), zeatin riboside (ZR), and dihydrozeatin (DHZ), are less active (Lomin et al. 2015). Recent findings suggested that ribotides can be directly converted into free-base forms by enzymes (Kudo et al. 2010). Active cytokinin levels are finely controlled by the enzymes that are involved in cytokinin biosynthesis and degradation. The homeostatic balance of cytokinin biosynthesis and catabolism mainly involves several enzymes, including isopentenyl transferase (IPT), cytochrome P450 monooxygenases (CYP75A), a cytokinin-activating enzyme (LONELY GUY, LOG), and a catabolic enzyme cytokinin oxidase/dehydrogenase (CKX) (Duan et al. 2019). In cytokinin synthesis, the initiation of iP and *t*Z biosynthesis catalyzed by IPT is also the rate-limiting enzyme (Kudo et al. 2010). Overexpression of* CKXs* reduces cytokinin levels and inhibits stem development (Ha et al. 2012). Overexpression of *AtCKX2* significantly reduces the levels of different intermediate metabolites of cytokinins (Werner et al. 2001). Moreover, the overexpression of *AtCKX5* may produce a more intense phenotype than other CKX genes (Ha et al. 2012). In rice, overexpression of *OsCKX4* or *OsCKX9* leads to shorter plant height and fewer grains. However, CRISPR/Cas9-generated *osckx9* was also shown to have a similar phenotype (Duan et al. 2019). Gene alterations in the cytokinin signaling pathway also affect plant height. For instance, the overexpression of the *type-A response regulator 6* (*OsARR6*) leads to reduced sensitivity to cytokinins and the development of dwarf phenotypes (Hirose et al. 2007; Gao et al. 2014). In tomato, *Solanum lycopersicum response regulator 6* (*SlRR6*) is an important component of the cytokinin, gibberellin, and indole-3-acetic acid (IAA) signaling networks that influence plant height (Liu et al. 2023).

Cytokinins are key factors in controlling cell division and cell cycle in plants. Changes in the concentration of cytokinins affect cell division rate and cell cycle (Riou-Khamlichi et al. 1999; Schaller et al. 2014). The cell cycle in plants is divided into G1, S, G2, and M phases, with G1/S and G2/M serving as important progression nodes (Qi and Zhang 2019). The regulation of cell division depends on cyclins, cyclin-dependent kinases (CDKs), and their related complexes. CYCBs are specifically expressed during the G2/M phase (Haga et al. 2011; Schaller et al. 2014). OsCYCB2;2 interacts with CDKA1 to regulate cell cycle (Peng et al. 2014). In addition to the classic CYCA, CYCB, and CYCD families, a new type of cyclin has been identified, the P-type cyclin (CYCP, also known as CYCU) (Torres Acosta et al. 2004; La et al. 2006; Chevalier 2008; Deng et al. 2014; Chen et al. 2020). OsCYCP4 integrates phosphate starvation signal with cell division (Xu et al. 2020). Under low-phosphorous conditions, *OsCYCP1;1* negatively regulates root growth (Deng et al. 2014). *CYCP2;1* is a target of WOX9, which is required to activate meristem growth during the germination of *Arabidopsis thaliana*. Overexpression of *CYCP2;1* rescues the short-stalk phenotype of *wox9* (Polyn et al. 2015). Brassinosteroids promote mesocotyl elongation via *CYCU2*-regulated cell division (Sun et al. 2018).

Gibberellins are a class of diterpenoids, that are biosynthesized via complex pathways. According to recent studies, gibberellins are believed to have a significant impact on the height of rice plants (Itoh et al. 2002; Sasaki et al. 2002), panicle development (Su et al. 2021), and nitrogen fertilizer efficiency (Camut et al. 2021). GA_1_, GA_3_, GA_4_, and GA_7_ are the main bioactive gibberellins. The first two stages of gibberellin synthesis are catalyzed by *CPS, KS, KO2*, and *KAO*. Subsequently, the branches form different gibberellin intermediates (Yamaguchi 2008). Research has found that genetic variations associated with gibberellin-synthesis result in stunted plant growth (Sakamoto et al. 2004). Gibberellin contents can vary after the overexpression or mutation of various genes, leading to either shortening or increased cell length (Lo et al. 2008; Chen et al. 2015; Zheng et al. 2018). In pea plants with strigolactone (SL)-related mutations, SL may stimulate cell division to increase stem elongation, in contrast to increasing cell length. SL appears to act independently of gibberellins to stimulate stem growth (de Saint Germain et al. 2013).

Different phytohormones function together rather than in isolation to control growth and development. Studies have shown that *knotted1-like homeobox* (*KNOX*) genes mediate gibberellin–cytokinin crosstalk (Jasinski et al. 2005; Wu et al. 2016; Su et al. 2021). *KNOX* gene expression promotes cytokinin signaling and inhibits gibberellin signaling (Jasinski et al. 2005). Overexpression of *knotted1* can increase cytokinin content and delay senescence (Ori et al. 1999). In potatoes, overexpression of *potato homeobox 1* (*POTH1*) inhibits gibberellin synthesis and alters vegetative development (Rosin et al. 2003). Furthermore, the *KNOX* gene family plays an essential role in the regulation of organogenesis and plant morphogenesis. Overexpression of *Oryza sativa homeobox 71* (*OSH71*) or *Oryza sativa homeobox 15* (*OSH15*) shows a reduction in the culm length, blade length, and panicle axis (Postma-Haarsma et al. 2002). *osh15* also shows defects in internode elongation and the development of epidermal and hypodermal cell types (Sato et al. 1998). *Oryza sativa homeobox 59* (*HOS59*) overexpression lines have lower plant height and smaller grain size (Sheng et al. 2022).

NAC (NAM, ATAF1/2, and CUC2) transcription factors are involved in multiple aspects of plant development and growth. Many genes have been shown to regulate plant height development. *OsNAC2* regulates the response of gibberellins, cytokinins, and auxins to affect plant height and root development (Mao et al. 2020). *XND1 (ANAC104)* regulates lignocellulose synthesis and programmed xylem cell death, thereby affecting plant height (Zhao et al. 2008). *OsNAC6* improves drought resistance by increasing the number and diameter of plant roots (Lee et al. 2017). However, the functions of more NAC family genes warrant investigation. Although the previous reports have found that gibberellins, drought, and low-temperature conditions can affect the expression level of *OsNAC103*, the other functions of *OsNAC103* that regulate plant development still remain to be investigated (Jeong et al. 2010; Nuruzzaman et al. 2010, 2012, 2015).

In this study, we constructed transgenic plants and conducted a series of experiments to elucidate the regulation of *OsNAC103* on plant growth. We measured the phytohormone contents to determine which phytohormones were affected by *OsNAC103*. To identify the genes and pathways involved in the plant height regulation of *OsNAC103*, we treated the seedlings with different phytohormones and detected the expression levels of related genes.

## Materials and methods

### Generation and cultivation of plant materials

To construct the *OsNAC103* overexpression vector, the coding DNA sequence (CDS) of *OsNAC103* was obtained from the leaf cDNA library of Taipei 309 (TP309, a *japonica* rice cultivar) and fused to pCXUN, which is an overexpression vector driven by the maize ubiquitin promoter. For β-glucosidase (GUS) staining, the 3981 bp genomic fragment upstream of ATG was amplified from wild-type (WT, TP309) genomic DNA and inserted into the GUS reporter vector pCXGUS. The target gene interference fragment was recombined with the interference vector pH7GWIWG2 (II) to construct the RNA interference (RNAi) vector. The vectors were transformed into TP309 calluses using the *Agrobacterium strain* EHA105 to obtain transgenic plants. Vector information for pCXUN and pCXGUS has been published previously (Chen et al. 2009). CRISPR/Csa9 mutant plants were obtained from BIOGLE Gene Tech Co., Ltd. (Jiangsu, China). The mutant rice variety used was Zhonghua 11 (ZH11, a *japonica* cultivar). The seedlings were grown in a culture room at 28 °C/25 °C (day/night). Seeds were obtained from rice plants grown under natural conditions in a field in Xiamen.

### Microscopical observation

Cell length was measured in the middle of the second leaf sheath of the 21-day-old seedlings. Samples were soaked in ethanol for decolorization and photographed using a Leica DM4B microscope.

### GUS staining

Samples were obtained from *proOsNAC103:GUS* transgenic plants. First, the samples were placed in a precooled 90% acetone solution for 30 min. After being washed thrice with pre-chilled ddH_2_O, the samples were incubated in a staining solution (50 mM sodium phosphate, (pH 7.2), 2 mM K_3_Fe(CN)_6_, 2 mM K_4_Fe(CN)_6_, 0.2% [v/v] Triton X-100, and 2 mM X-Gluc) for overnight at 37 °C. The samples were soaked in ethanol several times to rinse, decolorize, and remove the chlorophyll. The images were captured using a Leica M165 FC microscope.

### Subcellular localization analysis of OsNAC103

To investigate the subcellular distribution of OsNAC103, the CDS of *OsNAC103* was cloned and inserted into the pXDG vector to generate the *35S::GFP-OsNAC103* vector. The nuclear localization signal (NLS) sequence was cloned and inserted into the PXDR vector to obtain the vector *35S::RFP-NLS* as a nuclear marker. The two vectors were transferred together into rice protoplasts for transient expression. The preparation and transformation of rice protoplasts were performed according to the methods described by Jiang et al. (2018). Fluorescence signals in the protoplasts were visualized using a confocal microscope (Zeiss, LSM780). Vector information for pXDG and pXDR has been published previously (Chen et al. 2009).

### qRT-PCR analysis

An Eastep Universal RNA Extraction Kit (Promega) was used to extract total RNA. Promega GoScript was used to perform reverse transcription. qRT-PCR was performed using the SYBR Green Master Mix (Yeasen, Wuhan, China) in a LightCycler 480 system. qRT-PCR is performed as follows: 95 °C for 5 min, followed by 40 cycles at 95 °C for 5 s and 60 °C for 30 s. Three technical replicates and three biological replicates were prepared for every gene. The internal control was rice *actin 1*.

### Treatment of plant materials

To measure the induced expression levels of *OsNAC103* under various phytohormone treatments, WT plants were cultivated on 1/2 Murashige and Skoog (MS) medium. The seedlings at 21 days were treated with 100 µM N^6^-benzyladenine (6-BA) and 100 µM iP. Leaves were harvested at different time points after treatment. To evaluate phytohormone sensitivity, different plants were germinated and transferred to 1/2 MS alone or 1/2 MS with various concentrations of phytohormones (1 µM, 10 µM GA_3_; 1 µM, 10 µM 6-BA; 1 µM, 10 µM iP) or 10 µM paclobutrazol (PAC, a synthetic inhibitor of gibberellin) as the treatments for 10 days. For dark-induced stress experiments, leaves were subjected to 0, 2, 3, or 4 days without light to assess yellowing.

### Yeast two-hybrid assay and yeast one-hybrid assay

The yeast two-hybrid assay was used to confirm whether OsNAC103 has transactivation activity. The OsNAC103 protein sequence was divided into two parts based on the NAM domain, one containing the protein sequence from 1 to 139 amino acids (OsNAC103^△C^) and the other containing the protein sequence from 140 to 346 amino acids (OsNAC103^△N^). The full-length OsNAC103 sequence and truncated sequence were cloned and inserted into the vector GAL4-BD (pGBKT7; Clontech). The fused vectors and GAL4-AD (pGADT7; Clontech) were cotransformed into the yeast strain Y2HGold. The yeast transformation protocol was based on the Clontech Yeast Two-Hybrid System. Co-transformant with pGBKT7-53 and pGADT7-T was used as a positive control. Co-transformant with pGBKT7-Lam and pGADT7-T was used as a negative control. The conserved domain range of the NAM domain of OsNAC103 was based on the Rice Genome Annotation Project Database.

To perform the yeast one-hybrid assay, the CDS region of *OsNAC103* was fused to the vector pB42AD. Then, the construct was cotransferred into the yeast strain EGY48 with the *LacZ* reporter vector (pLacZi2µ) driven by the promoter of *OSH71* (1774 bp upstream from ATG). The transformants were examined on SD/− Ura/− Trp plates and were chromogenic on plates containing X-gal. Co-transformant with pB42AD-HY5 and proCOP1 was used as a positive control.

### Dual-luciferase assay in rice protoplasts

To assess the transcriptional activity of OsNAC103, the CDS of *OsNAC103* was linked to the effector vector (pXSN) driven by the *35S* promoter. The promoter of *OsCYCP2;1* (1500 bp upstream from ATG) was fused to a reporter vector (pGreenII 0800-LUC). Different carrier combinations were co-transfected into the rice protoplasts in a ratio of 1:1. The preparation and transformation of rice protoplasts were performed according to the methods described by Jiang et al. (2018). The protoplasts were collected for dual-luciferase measurements. The detailed experimental methods for the Promega Dual-Luciferase Reporter Assay System are described in the manufacturer's instructions. The transcriptional activity levels were calculated using the relative LUC/REN ratio.

### Measurements of phytohormones

To quantify phytohormones, the leaves of 21-day-old WT and *OE-OsNAC103* seedlings were harvested. Each sample consisted of three biological samples. The phytohormone contents were analyzed at the Shanghai Applied Protein Technology Co., Ltd. (Shanghai, China). The samples were ground with liquid nitrogen, and samples weighing 100 ± 5 mg were placed in 2 mL centrifuge tubes. To ensure complete extraction, 30 μL of internal standard solution and 1.17 mL of acetonitrile were added to the 2 mL centrifuge tubes. The solution was vortexed until sufficiently mixed. The resulting mixture was ultrasonicated for 25 min at low temperature and avoiding light and then allowed to stand overnight at − 20 °C. After centrifugation (14,000 g, 4 °C, 20 min), the resulting supernatants were subjected to filtration and subsequently evaporated under N2 until dryness. Before mass spectrometry analysis, the extracts were dissolved again in 200 μL of a mixture of methanol and water (1:1, v/v). An AB SCIEX system was used for the mass spectrometry analysis, with the system set to the positive/negative ionization mode.

### Electrophoretic mobility shift assay (EMSA)

The EMSA reaction system was as follows: 0.02% BSA, 8% glycerol, 0.5% Triton X-100, 10 × EMSA Binding Buffer [10 mM MgCl_2_, 200 mM KCl, 10 mM DTT, and 100 mM Hepes (pH = 7.8)], probe, purified GST-NAC103 protein or GST, and 300 ng of salmon essence, to which ddH_2_O was added to bring the volume up to 20 μL. The solution was thoroughly mixed and incubated for 30 min in the dark at room temperature. The probes were labeled with Texas Red. The EMSA reaction products were resolved on a 6% native polyacrylamide gel in TBE buffer. After electrophoresis, a Bio-Rad instrument and Texas Red filter were used for exposure.

## Results

### Comparative analysis of the OsNAC103 protein and its homologs

**Fig. 1:**
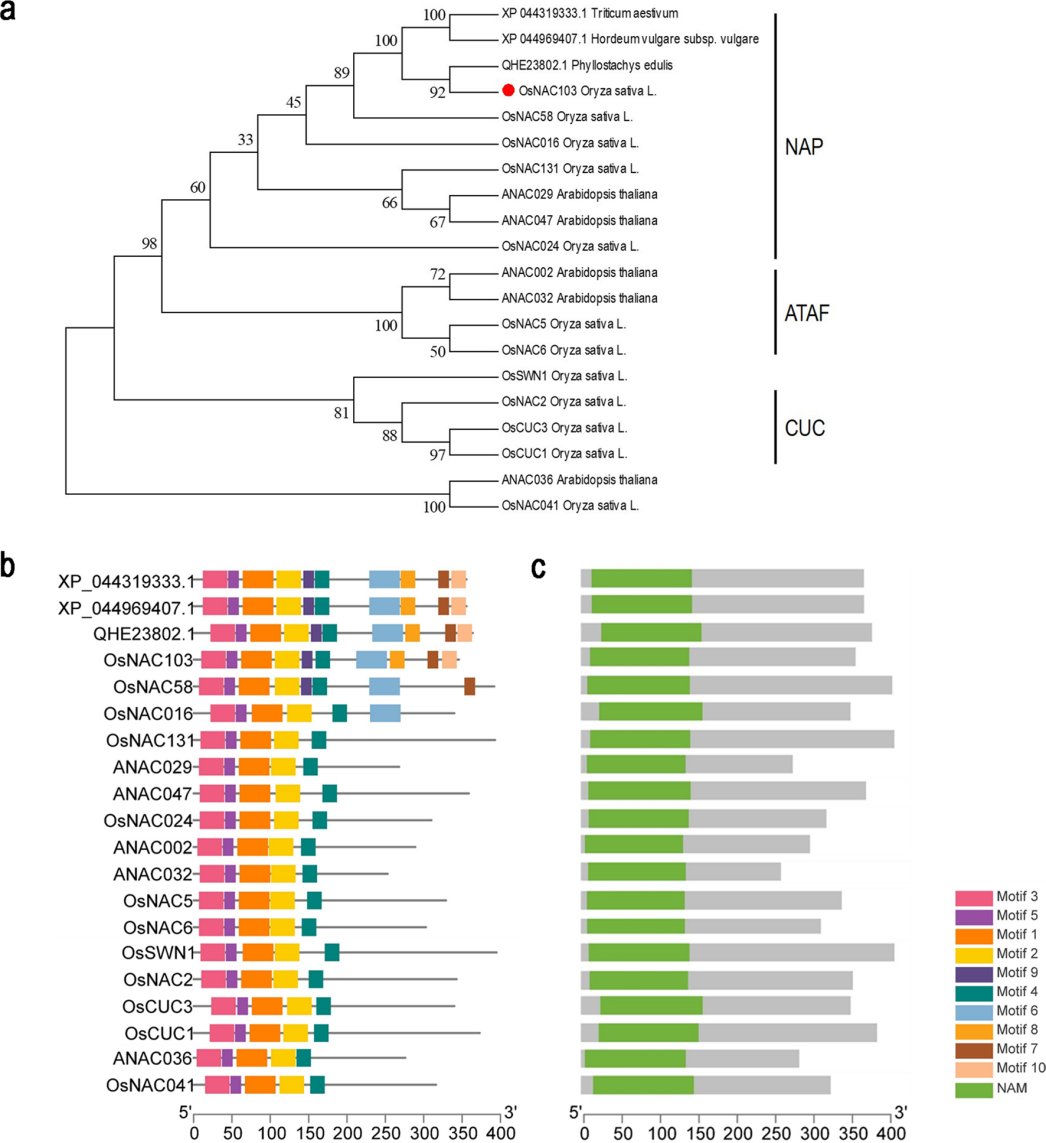
Phylogenetic tree and conserved sequence analysis of OsNAC103 proteins homologs. **a** Phylogenetic tree analysis of protein homologs of OsNAC103. The neighbor-joining (NJ) phylogenetic tree was constructed using MEGA5. **b** Conserved motif analysis of OsNAC103 and homologous proteins. The MEME program was used to investigate the conserved motifs. The motif width was set from 6 to 200. The motif number was set to 10. Differently colored rectangles represent different domains. **c** The green label shows the location of the NAM conserved domains of OsNAC103 and homologous proteins

The *OsNAC103* gene encodes a protein of 346 amino acids, that belongs to the NAC transcription factor family. OsNAC103 is a member of the NAP (NAC-Like, Activated by AP3/PI) subfamily, which includes seven rice NAP proteins (Fan et al. 2015). OsNAC58 and OsNAC131 (in the RAP-DB rice database), ANAC029 (ATNAP), and ANAC047 (in the TAIR Arabidopsis database) were highly homologous to OsNAC103. All these genes belong to the NAP family. The protein homologs of OsNAC103 from other species were screened using BLAST from the NCBI database. It was found that OsNAC103 had the highest similarity with QHE23802.1 (*Phyllostachys edulis*), followed by XP_044969400.7 (*Hordeum vulgare*) and XP_044319333.1 (*Triticum aestivum*). The above genes and some reported NAC transcription factors involved in plant development were analyzed by constructing a phylogenetic tree. These findings indicated that OsNAC103 clustered with other NAP family genes (Fig. 1a). Studies have shown that plants overexpressing *OsNAC58* exhibit an obvious phenotype of yellowing and senescence (Liang et al. 2014), suggesting that the *OsNAC103* gene may function similarly to *OsNAC58*.

Researches show that NAC family proteins have a highly conserved NAC domain in their N-terminal regions and a variable C-terminal domain that functions as a transcriptional activation region (Ooka et al. 2003; Puranik et al. 2012). MEME was used for the motif analysis of all proteins in the phylogenetic tree. The conserved motifs are labeled in Fig. 1b, and ten motifs were screened. The NAM domains of the corresponding NAC transcription factor families in the phylogenetic tree are shown in Fig. 1c. Motifs 1–5 represent the conserved subdomains of the N-terminus of NAC transcription factors. OsNAC103 also has a motif 9 at its N-terminus. Compared to other genes, the NAP subfamily has more conserved sequences in the C-terminal region. In the NAP subfamily, in addition to OsNAC16, OsNAC131, ANAC029, and ANAC047, other proteins, including OsNAC103, also contain motif 8, motif 7, and motif 10 in the C-terminal transcriptional activation region. These differences may indicate that this protein has additional functions in plant growth and development.

### OsNAC103 subcellular localization and expression pattern analysis

**Fig. 2:**
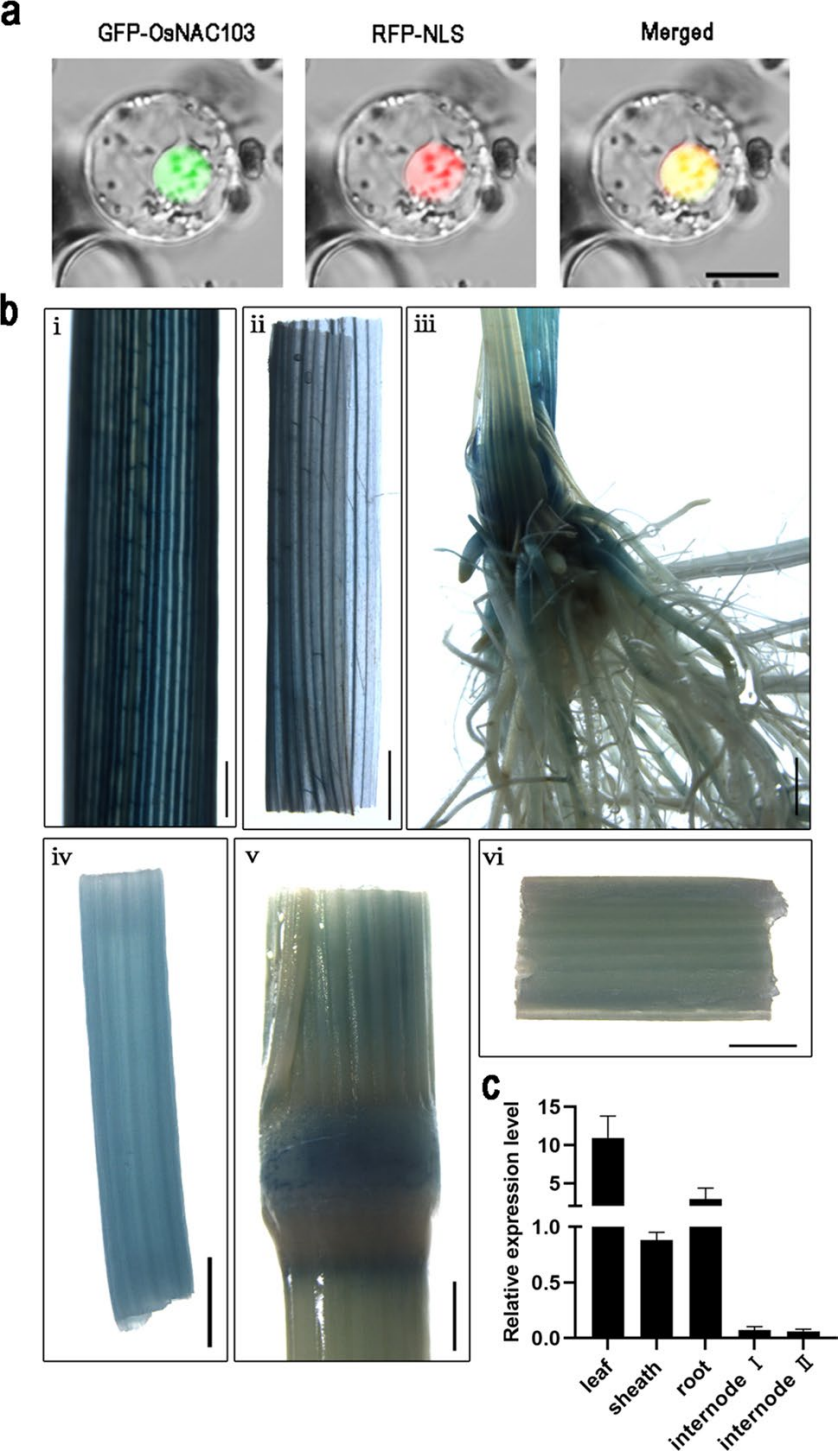
OsNAC103 subcellular localization and tissue expression analysis. **a** The subcellular localization of OsNAC103. Bar = 10 μm. RFP-NLS was used as a nuclear marker. **b** GUS activity was detected in the young leaf (**i**), the young leaf sheath (**ii**), the young root (**iii**), the first internode (**iv**), the stem node (**v**), and the inner wall of the second internode of rice at maturity (**vi**). Bar = 1 mm. **c** Relative *OsNAC103* expression levels in different tissues. Mean values ± SD, *n* = 3. Leaf, sheath, and root of 21-day-old seedling; internode I and internode II: the first internode and the second internode of mature plants

To further study the function of *OsNAC103*, a *35S::GFP-OsNAC103* vector was constructed and transferred to rice protoplasts for fluorescence observation. Subcellular localization analysis showed that OsNAC103 was mainly located in the nucleus, colocalizing with the signal of RFP fused with the nuclear localization signal (NLS; Fig. 2a). In terms of the expression pattern of *OsNAC103,* the analysis based on MBKbase revealed that *OsNAC103* showed spatiotemporal expression at various stages of rice development (Fig. S1). To directly observe the tissue expression of *OsNAC103*, the gene promoter was inserted into a vector that included the GUS reporter and then transformed to obtain transgenic plants. GUS activity was detected in leaves, leaf sheaths, and roots (Fig. 2b i–iii). Furthermore, weaker GUS staining was detected in the first internode, stem node, and inner wall of the second internode (Fig. 2b iv–vi). qRT-PCR was performed on tissues during the seedling and mature stages. The highest expression level of *OsNAC103* was observed in the leaves of 21-day-old seedlings, followed by the roots and leaf sheaths, and was the lowest in the internodes (Fig. 2c). These findings indicate that *OsNAC103* may play a role in leaf and stem development.

### Identification of transcriptional activation of the OsNAC103 protein

**Fig. 3:**
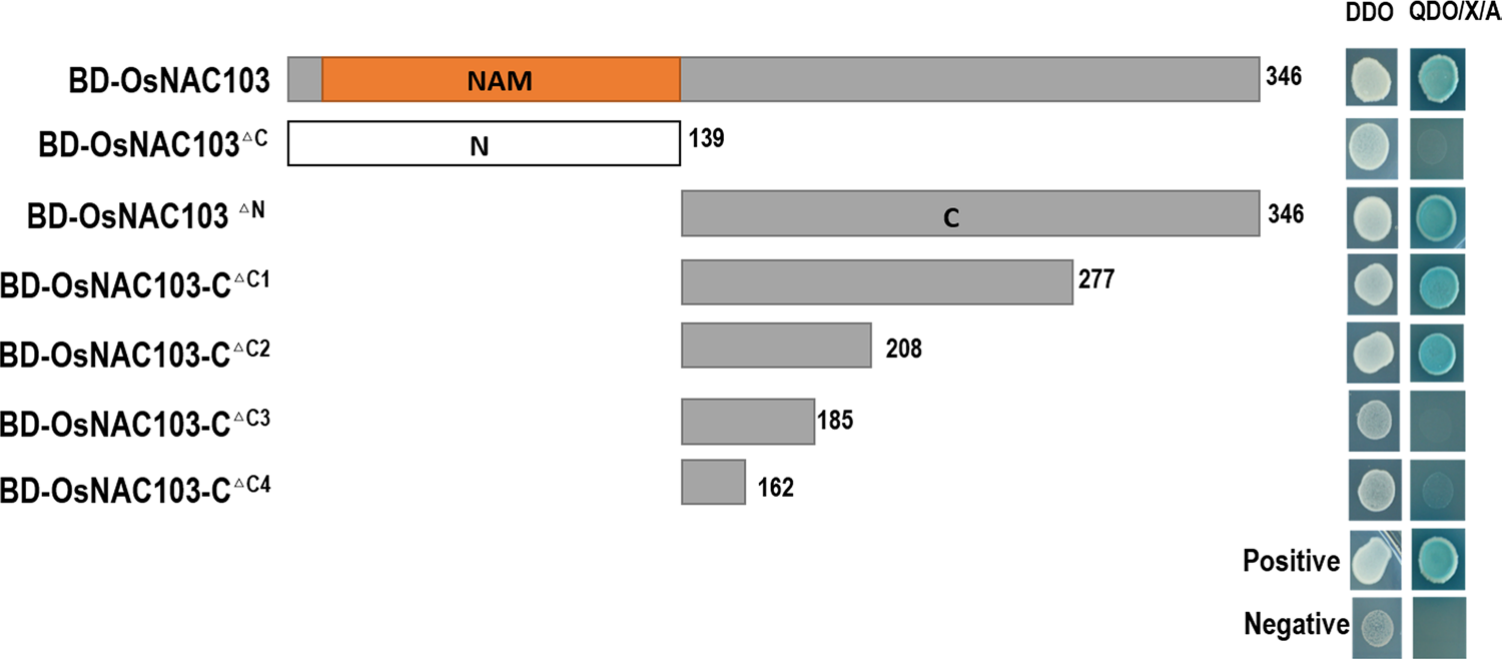
Transactivation activity of the OsNAC103 protein. The positive control: pGBKT7-53 and pGADT7-T plasmids. The negative control: pGBKT7-Lam and pGADT7-T plasmids

To ascertain whether OsNAC103 has transcriptional activation activity, the OsNAC103 protein was fused to GAL4-BD. The results showed that yeast transformed with the full-length fusion protein BD-OsNAC103 could grow on the QDO/X/A medium, indicating that OsNAC103 had self-activation activity. Then, the OsNAC103 protein sequence was divided into two segments based on the location of the NAM domain. One contained the protein sequence from 1 to 139 amino acids (OsNAC103^△C^), while the other contained the protein sequence from 140 to 346 amino acids (OsNAC103^△N^). However, only yeast cells harboring BD-OsNAC103^△N^ grew, while yeast cells carrying BD-OsNAC103^△C^ did not grow on the QDO/X/A medium. These results indicate the transcriptional activation region at the C-terminus (Fig. 3). To investigate whether a specific portion of OsNAC103^△N^ is the determining factor for transactivation activity, we further performed truncated experiments. Notably, the 186‒208 amino acid region may play a prominent role in regulating the transactivation activity of OsNAC103.

### OsNAC103 negatively regulates plant height

To explore the function of *OsNAC103* in rice, *OsNAC103*-overexpressing transgenic lines and RNA interference (RNAi) lines were constructed in the TP309 background, and CRISPR/Csa9 mutants were constructed in the ZH11 background.

**Fig. 4:**
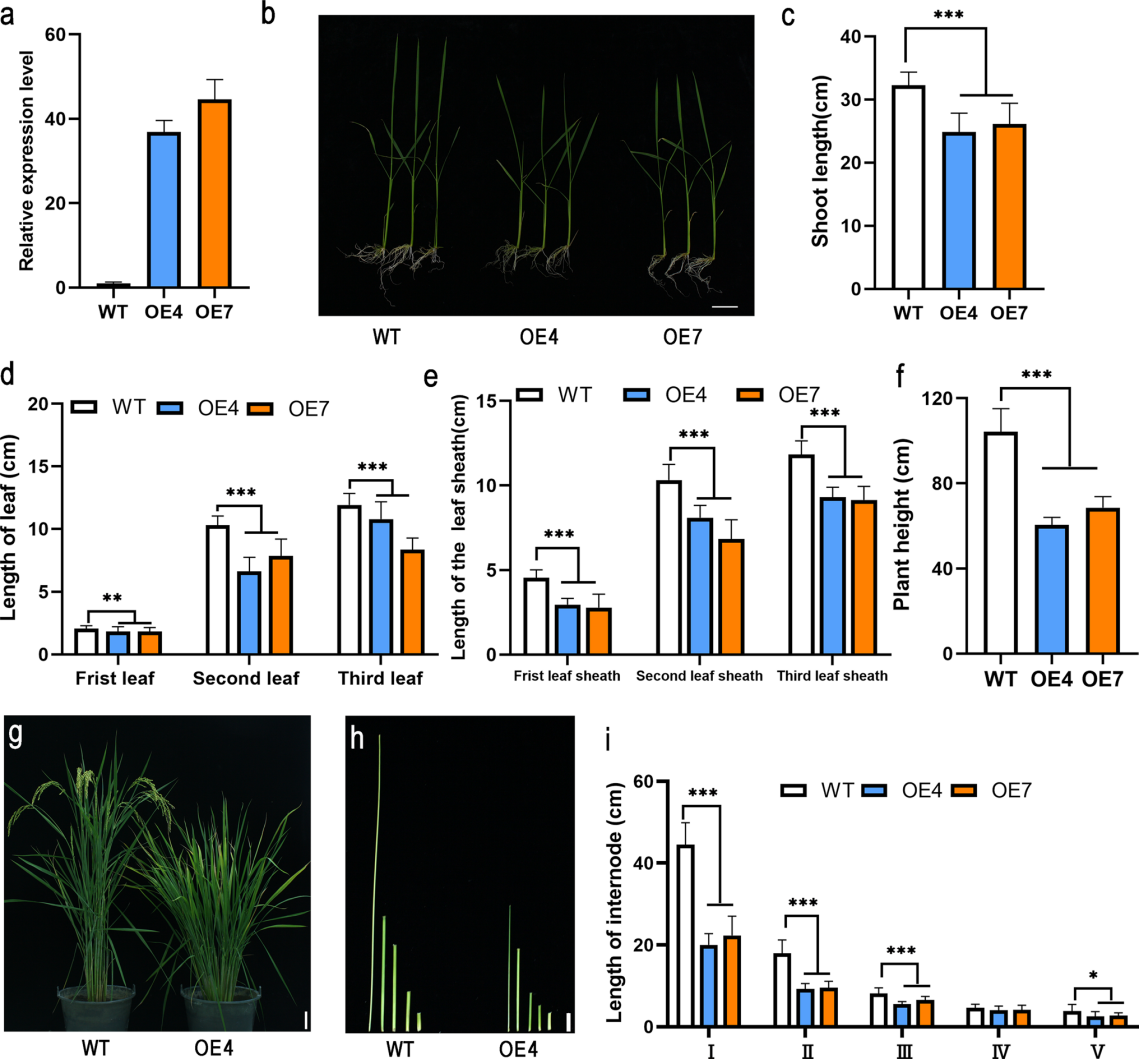
Phenotypes of *OE-OsNAC103* plants. **a** The relative expression level of *OsNAC103* of 21-day-old WT and *OE-OsNAC103* plants. Mean values ± SD,* n* = 3. **b** The phenotype of 21-day-old WT and *OE-OsNAC103* plants*.* Bar = 2 cm. **c** The shoot length of 21-day-old WT and *OE-OsNAC103* plants. Mean ± SD, *n* = 20. **d** The leaf length of 21-day-old WT and *OE-OsNAC103* plants. Mean ± SD, *n* = 25.** e** The leaf sheath length of of 21-day-old WT and *OE-OsNAC103* plants. Mean ± SD, *n* = 28. **f** The plant height of mature WT and *OE-OsNAC103* plants. Mean ± SD, *n* = 10. **g** The phenotype of mature-stage WT and *OE-OsNAC103* plants. Bar = 5 cm. **h** The different internodes of mature WT and *OE-OsNAC103* plants. Bar = 2 cm.** i** The internode lengths of mature WT and *OE-OsNAC103* plants (from the top of the stem to the bottom). Mean ± SD, *n* = 13. The WT was used as a control for significance difference analysis. *P < 0.05; **P < 0.01; ***P < 0.001; *t* test

**Fig. 5:**
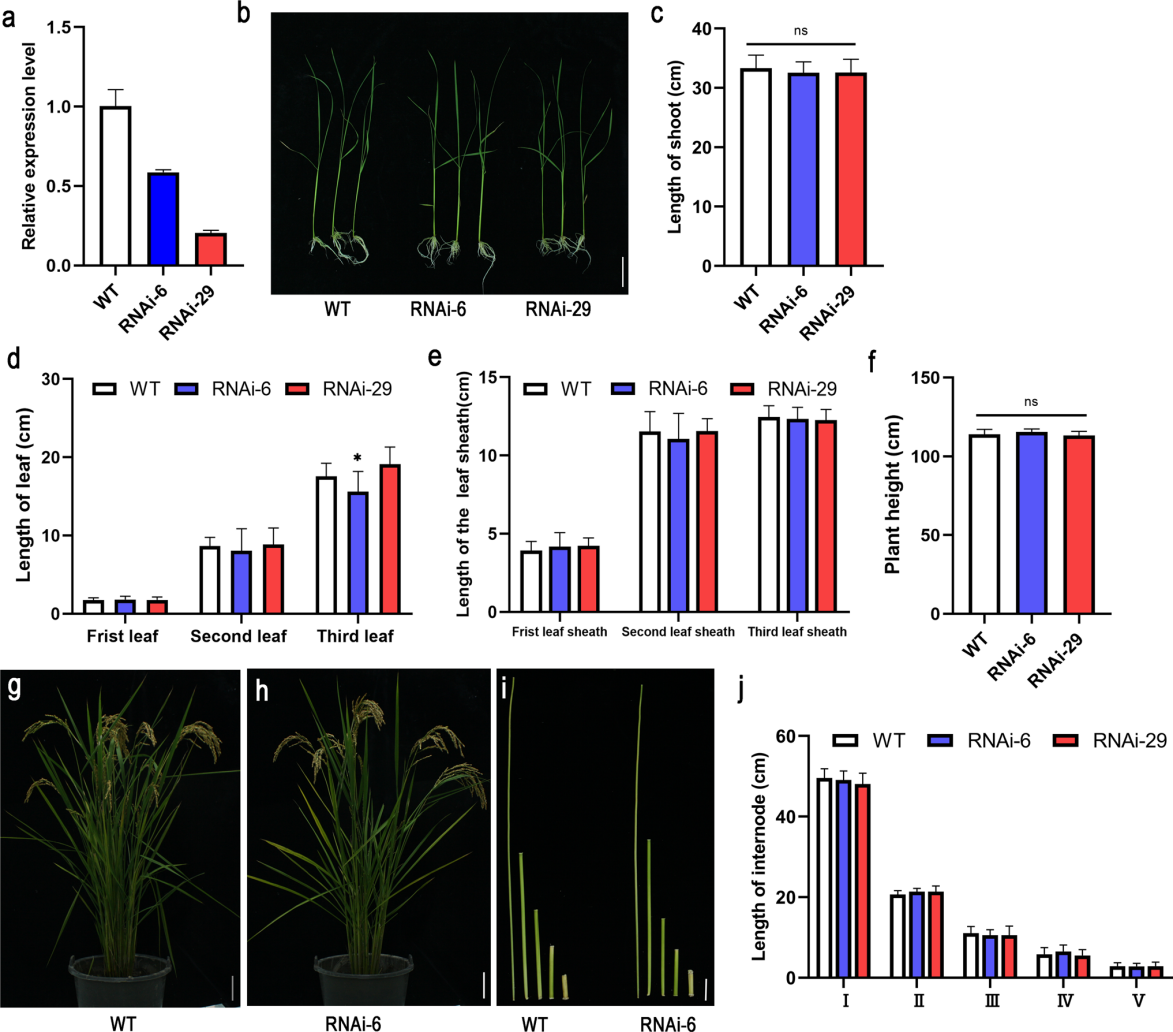
Phenotypes of *RNAi*-*OsNAC103* plants. **a** The relative expression level of *OsNAC103* of 21-day-old WT and RNAi lines. Mean values ± SD, *n* = 3. **b** The phenotype of 21-day-old WT and RNAi plants. Bar = 5 cm. **c** The shoot length of 21-day-old WT and RNAi plants. Mean ± SD, *n* = 15.** d** The leaf length of 21-day-old WT and RNAi plants. Mean ± SD, *n* = 15. **e** The leaf sheath length of 21-day-old WT and RNAi plants. Mean ± SD,* n* = 15.** f** The plant height of mature WT and RNAi plants. Mean ± SD, *n* = 8. **g** The phenotype of mature-stage WT. Bar = 10 cm.** h** The phenotype of mature-stage RNAi plants. Bar = 10 cm. **i** The different internodes of mature WT and RNAi lines. Bar = 3 cm.** j** The internode lengths of the WT and RNAi plants (from the top of the stem to the bottom). Mean ± SD, *n* = 8. The WT was used as a control for significance difference analysis. *P < 0.05; ns, no significant difference, *t* test

*OE-OsNAC103* plants exhibited a dwarf phenotype at the 21-day-old seedling stage (Fig. 4a, b). The lengths of the shoots, leaves, and leaf sheaths in *OE-OsNAC103* seedlings were shorter than the corresponding values of the WT (Fig. 4c–e). Nevertheless, no notable differences were observed between the RNAi lines and the WT (Fig. 5a–e). At the heading stage, slower vegetative growth and lower plant height than those of the WT were observed in the OE4 and OE7 lines (Fig. 4f, g). The length of the internodes was reduced relative to that of the WT (Fig. 4h, i). Even during rice maturation, the RNAi plants did not exhibit notable variations in plant height (Fig. 5f–j). In addition, the phenotype of the *osnac103* mutant also exhibited no notable disparity in plant height compared with that of WT (ZH11) (Fig. S2). Taken together, these results revealed that *OsNAC103* negatively regulates plant height and development.

### *OsNAC103* regulates gibberellin metabolism but does not affect cell length

**Fig. 6:**
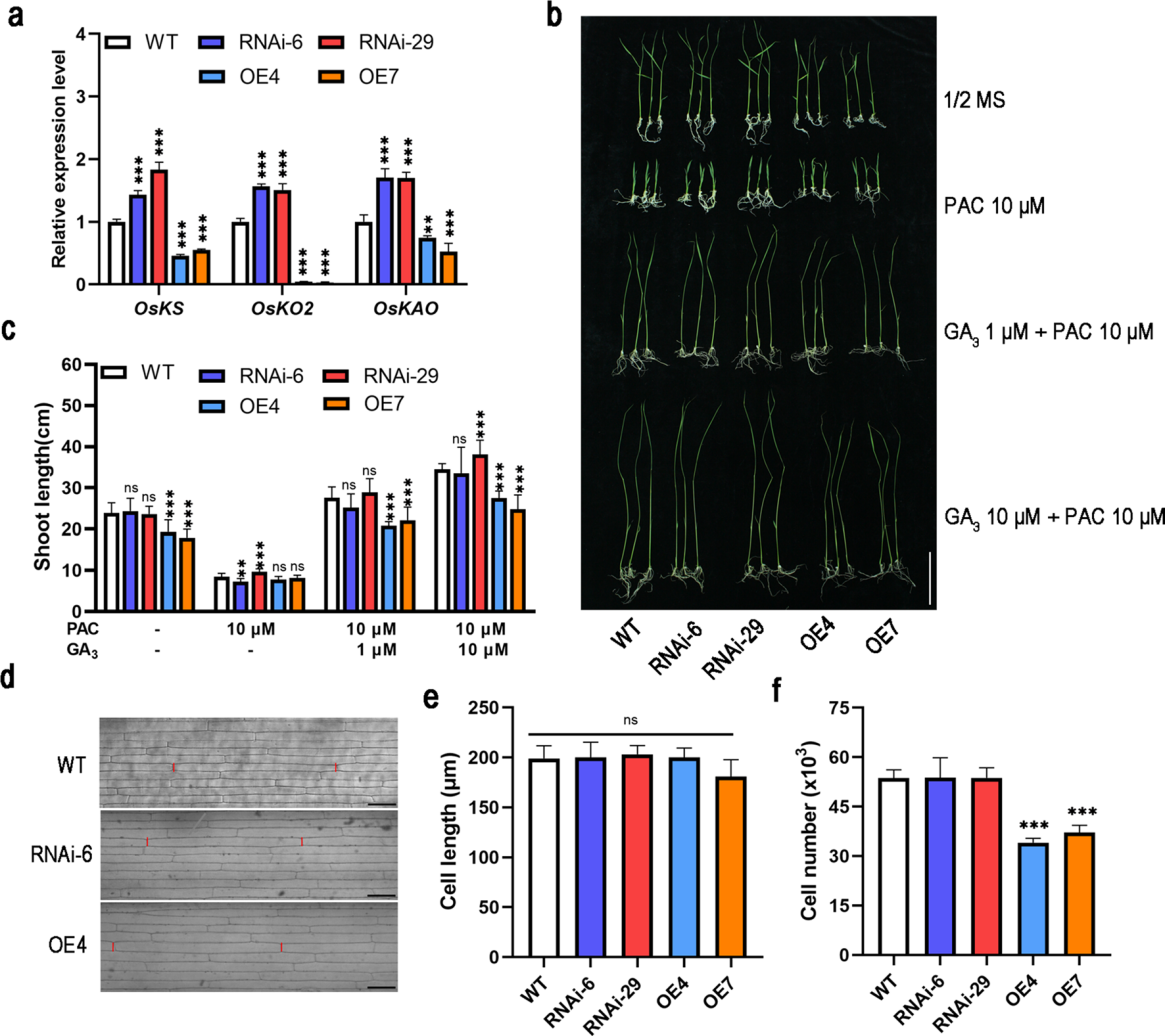
*OsNAC103* regulates gibberellin metabolism and cell numbers. **a** The relative expression level of genes related to gibberellin synthesis in WT, RNAi, and *OE-OsNAC103* plants. Mean values ± SD, *n* = 3. **b** The phenotype of WT, RNAi, and *OE-OsNAC103* plants incubated in 1/2 MS medium or medium-containing PAC, GA_3_ for 10 days. Bar = 5 cm. **c** The shoot length of WT, RNAi, and *OE-OsNAC103* plants incubated in 1/2 MS medium or medium-containing PAC, GA_3_ for 10 days. Mean ± SD, *n* = 10.** d** The epidermal cells in the second leaf sheath of 21-day-old seedlings. Bar = 50 μm. **e** The cell length of WT, RNAi, and *OE-OsNAC103* plants. Mean ± SD. Every line has at least 300 cells.** f** Estimation of cell numbers in the second leaf sheath of 21-day-old seedlings

A previous study showed that gibberellins are the main factors determining plant height (Salas Fernandez et al. 2009). Therefore, we investigated the relationship between the dwarf phenotype and gibberellins. The results showed that the expression of the gibberellin biosynthesis-related genes *OsKS*, *OsKO2*, and *OsKAO* were suppressed in *OE-OsNAC103* plants, but increased in RNAi lines (Fig. 6a). These results suggested that gibberellin synthesis was negatively regulated. We examined the growth of the WT, RNAi lines, and *OE-OsNAC103* plants treated with PAC and exogenous GA_3_. After 10 days of 10 μM PAC treatment, both the WT and transgenic lines were significantly inhibited, and the difference in plant height was dramatically reduced. The plant height increased (compared to normal growth) after the application of 1 μM GA_3_ and 10 μM GA_3_. In contrast, the plant height of the RNAi lines was similar to that of the WT except in plants treated with 10 μM GA_3_. However, plant height remained lower in the overexpressing plants than in WT plants and RNAi lines (Fig. 6b, c). These results indicated that the increased expression of *OsNAC103* decreases sensitivity to gibberellins.

Gibberellins exert effects on growth by altering cell length (Thingnaes et al. 2003; Chen et al. 2015). Accordingly, we compared the cell lengths of the second leaf sheaths, and the statistical analysis showed no significant differences (Fig. 6d, e). This suggests that dwarfing is not caused by changes in cell length. Cell numbers were estimated using the ratio of leaf sheath length to cell length. The results showed that the overexpression of *OsNAC103* significantly reduces the number of cells (Fig. 6f). The decrease in cell numbers is the main cause of dwarfing.

### *OsNAC103* overexpression results in a reduction in cytokinin content

**Fig. 7:**
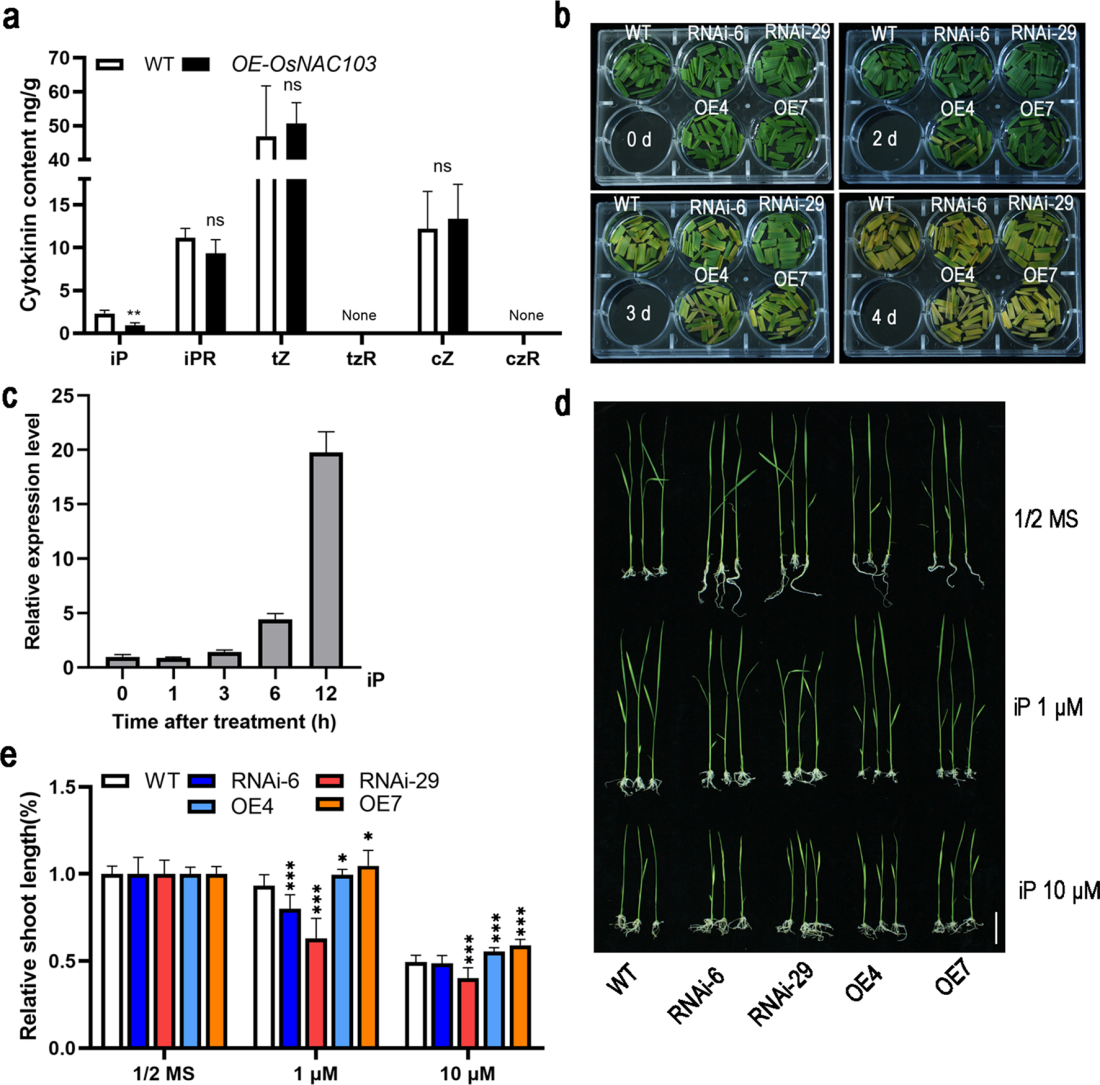
Overexpression of *OsNAC103* decreased the cytokinin content and sensitivity to iP. **a** The comparison of WT and *OE-OsNAC103* plants’ cytokinin content. Mean values ± SD, *n* = 3.** b** Observation of dark-induced leaf yellowing phenotypes in WT, RNAi, and *OE-OsNAC103* plants.** c** The relative expression level of *OsNAC103* under iP (100 µM) treatment in WT plants. Mean ± SD, *n* = 3.** d** Phenotypes of WT, RNAi, and *OE-OsNAC103* plants treated with different concentrations of iP for 10 days. Bar = 5 cm. **e** The relative shoot length of WT, RNAi, and *OE-OsNAC103* plants treated with different concentrations of iP for 10 days. Mean ± SD, *n* = 8. The WT was used as a control for significance difference analysis. **P < 0.01; ***P < 0.001; ns, no significant difference, *t* test

To confirm whether phytohormone levels were altered in *OsNAC103*-related dwarf plants, endogenous phytohormone levels were measured. This finding indicated a significant decrease in iP levels in *OE-OsNAC103* plants, which was only 40% of that in WT plants. In addition, there were no notable differences in the levels of other cytokinin components and other phytohormones (Fig. 7a, Fig. S3). Dark-induced stress experiments showed that cytokinins can delay chlorophyll degradation in mature green leaves (Zhang et al. 2021). Therefore, we conducted dark induction experiments on WT and transgenic plants. After 2 days of dark treatment, the leaves of *OE-OsNAC103* plants showed an early yellowing trend. On the third day of treatment, the WT and RNAi strains showed delayed leaf yellowing. The above results indicate that the decrease in cytokinin content was more likely to exhibit a yellowing phenotype (Fig. 7b). *OsNAC103* can affect plant growth by reducing the cytokinin content.

### Overexpression of *OsNAC103* decreased plant susceptibility to iP

In plants, iP is considered as one of the primary active forms of cytokinins. To further explore the relationship between *OsNAC103* and iP, the expression level of *OsNAC103* in plants treated with exogenous cytokinin iP was tested. As shown in Fig. 7c, upon exogenous iP treatment, the *OsNAC103* transcript level was significantly increased in the WT plants, indicating that *OsNAC103* participates in the regulation of iP response.

Then, the WT, RNAi, and *OE-OsNAC103* strains were treated with 1 µM iP and 10 µM iP. Compared with seedlings grown on 1/2 MS medium, the growth of plants was suppressed under 1 µM iP treatment, and the relative height of the RNAi plants was significantly lower than that of the WT. The relative plant height of the *OE-OsNAC103* plants was considerably higher than that of the WT. When treated with 10 μM iP, plants of all lines showed restricted growth. The RNAi strain exhibited a higher degree of inhibition than the *OE-OsNAC103* strain. The relative plant height of *OE-OsNAC103* was significantly higher than that of the WT and RNAi plants (Fig. 7d, e). Similarly, when the *osnac103* mutant was treated with iP, the phenotype of the *osnac103* plants was consistent with that of the RNAi lines (Fig. S4a). These results indicated that *OE-OsNAC103* plants were not sensitive to iP. Overexpression of *OsNAC103* not only reduced the cytokinin content but also reduced the sensitivity to cytokinins.

### *OsNAC103* regulates cytokinin synthesis, degradation, and signal transduction

**Fig. 8:**
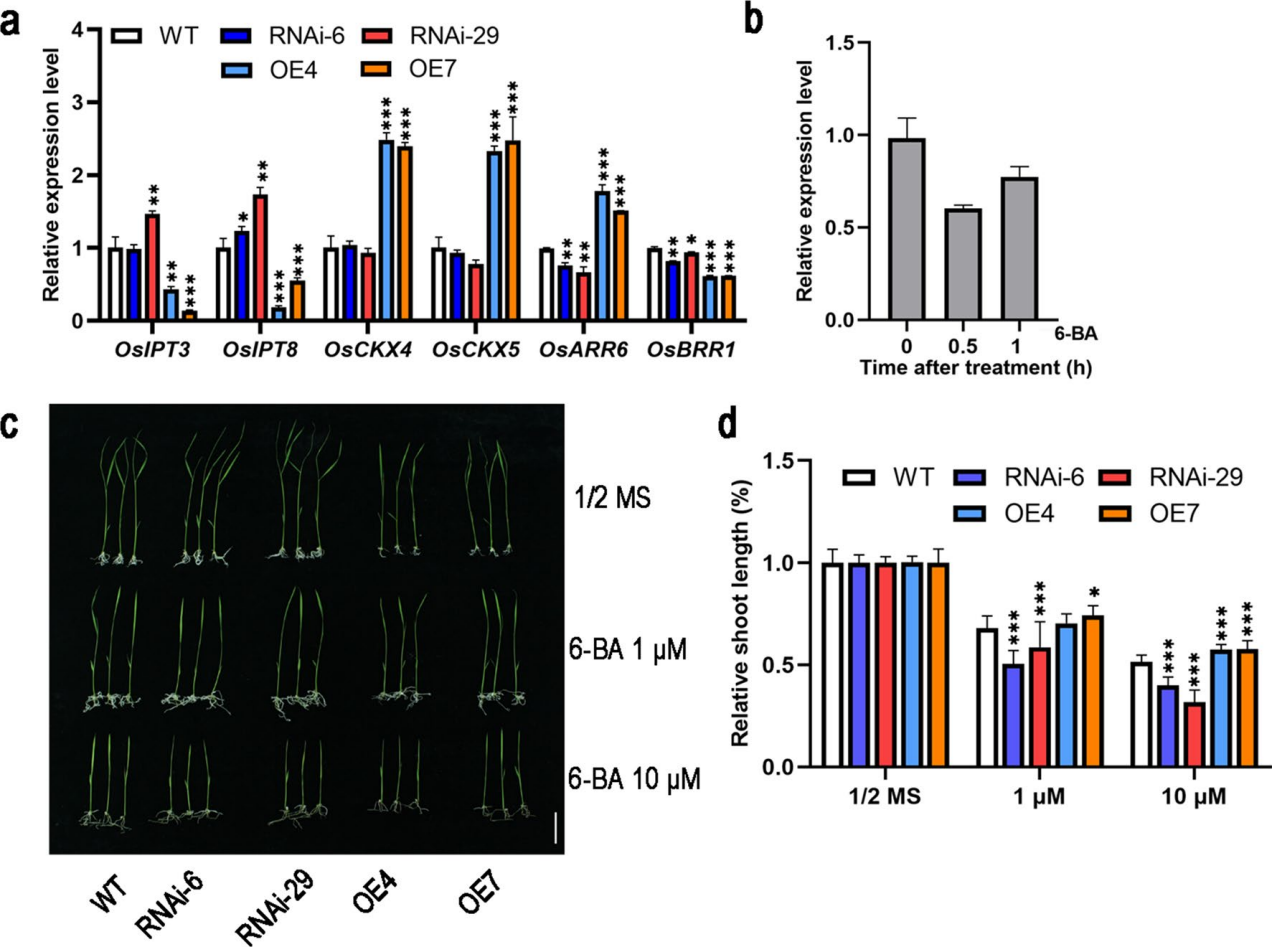
*OsNAC103* regulates cytokinin synthesis, degradation, and signal transduction. **a** The relative expression level of genes related to cytokinin synthesis, degradation, and signaling response in WT, RNAi, and *OE-OsNAC103* plants. Mean values ± SD, *n* = 3.** b** The relative expression level of *OsNAC103* in the WT under 6-BA (100 µM) treatment. Mean ± SD,* n* = 3. **c** Phenotypes of WT, RNAi, and *OE-OsNAC103* plants on 1/2 MS alone or treated with different concentrations of 6-BA for 10 days. Bar = 5 cm. **d** The relative shoot length of WT, RNAi, and *OE-OsNAC103* plants on 1/2 MS alone or treated with different concentrations of 6-BA for 10 days. Mean ± SD, *n* = 8. The WT was used as a control for significance difference analysis. *P < 0.05; **P < 0.01; ***P < 0.001; *t* test

Cytokinin levels are determined by the balance between their synthesis and metabolism. Because the iP content was decreased in *OE-OsNAC103*, we measured the expression levels of genes involved in cytokinin metabolism. As shown in Fig. 8a, the cytokinin biosynthesis-related genes *OsIPT3* and *OsIPT8* were activated in the RNAi plants but were inhibited in the overexpression plants. In contrast, *OsCKX4* and *OsCKX5* showed a more significant upregulation trend in *OE-OsNAC103* plants. Previous studies have shown that the CKX enzymes can irreversibly degrade iP (Zurcher and Muller 2016), and the enhanced CKX function promotes cytokinin degradation, leading to an increase in inactive cytokinins, thereby inhibiting plant growth and development. These results indicated that *OsNAC103* reduces bioactive cytokinin accumulation by promoting cytokinin degradation and inhibiting cytokinin synthesis.

To validate the connection between dwarfing and cytokinin, we investigated the response of *OsNAC103* transcripts to 100 μM 6-BA in WT leaves and found that the expression of *OsNAC103* was downregulated within 1 h (Fig. 8b). The sensitivity of the WT and transgenic lines to 6-BA was evaluated with 1 μM and 10 μM 6-BA. After treatment for 10 days, the plant height of both the WT and transgenic lines was restricted in comparison to that of plants cultivated under standard growth conditions. The inhibition rate was higher in the RNAi lines and *osnac103* plants than in the overexpression lines (Fig. 8c, d, Fig. S4b). These results indicate that the *OE-OsNAC103* plants were hyposensitive to 6-BA. To explore whether cytokinin signaling is affected, cytokinin response genes type-A and type-B RRs were analyzed. Type-A RRs negatively regulate cytokinin signaling, whereas type-B RRs are activators (To and Kieber 2008). The results showed that *OsARR6* was suppressed in the RNAi lines but increased in the overexpression lines. *OsBRR1* was downregulated (Fig. 8a). Thus, *OsNAC103* negatively regulates cytokinin biosynthesis and signaling pathways in rice.

### OsNAC103 represses cell cycle progression and *OsCYCP2;1* may act as a target gene

**Fig. 9:**
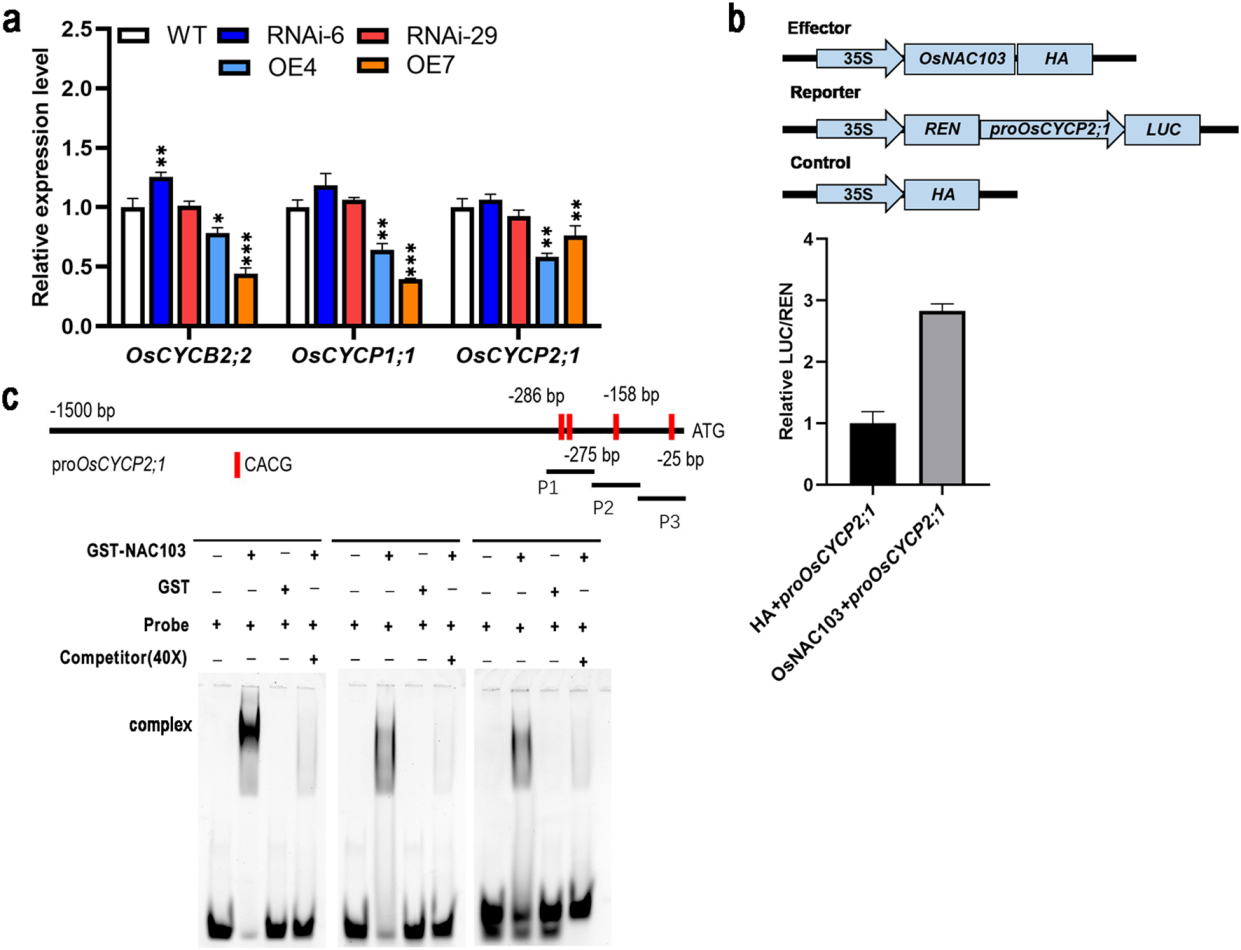
*OsNAC103* affects cell cycle progression genes. **a** The relative expression levels of cell cycle genes in WT, RNAi, and *OE-OsNAC103* plants. Mean values ± SD, *n* = 3. **b** Transactivation activity of OsNAC103 on the promoter of *OsCYCP2;1* was tested by dual-luciferase assay. Mean ± SD, *n* = 5. **c** A schematic diagram of the promoter of *OsCYCP2;1* and DNA-binding activities of OsNAC103 proteins on the CACG motifs of *OsCYCP2;1* was tested by EMSA. The WT was used as a control for significance difference analysis. *P < 0.05; **P < 0.01; ***P < 0.001; *t* test

Cytokinins can accelerate cell division, and there is a positive correlation between their levels and cell division (Yang et al. 2002). For example, *rice G-protein γ subunit* (*RGG1*) inhibits cell division by significantly reducing cytokinin biosynthesis, ultimately reducing plant height and panicle elongation (Tao et al. 2020). In *OsCKX3* overexpressing plants, the decrease in the expression of the cyclin-related gene *CYCP4;1* reduced cell proliferation (Huang et al. 2022). Because cell numbers were reduced in dwarf plants, we speculated that cell division might be inhibited in *OE-OsNAC103* plants. qRT-PCR analysis showed that *OsCYCP1;1, OsCYCP2;1*, and *OsCYCB2;2* were downregulated in the overexpression lines (Fig. 9a). Physical interactions between AtCYCP2;1 and CDKs affect the G2/M transition. Furthermore, *atcycp2;1* mutant plants exhibit seedling growth arrest (Torres Acosta et al. 2004; Peng et al. 2014). The findings indicated that *OsNAC103* hinders the regular progression of the cell cycle and restrains cell division, leading to a dwarf phenotype.

OsNAC103 is a transcription factor; therefore, it is important to identify its downstream regulatory target genes. We performed dual-luciferase experiments to determine whether *OsNAC103* regulates the promoter activity of cytokinin-related genes and cell cycle-related genes. It has been found that OsNAC103 can regulate the promoter activity of *OsCYCP2;1*. Compared to the control, the overexpression of *OsNAC103* led to a significant increase in the activity of the LUC reporter (Fig. 9b).

The CACG motif is a core-binding site for NAC transcription factors (He et al. 2015; Tang et al. 2019). There was more than one CACG motif in the *OsCYCP2;1* promoter (Fig. 9c). Therefore, we asked whether OsNAC103 could bind to the promoter of *OsCYCP2;1.* Next, the interaction between OsNAC103 protein and *proOsCYCP2;1* was tested by performing EMSA for further verification with purified OsNAC103 protein. These experiments confirmed the above results, showing that the OsNAC103 protein binds to the CACG motif in the promoter of *OsCYCP2;1* (Fig. 9c). Together, these data confirmed that *OsCYCP2;1* as a target of OsNAC103.

### *OsNAC103* affects plant development by regulating *KNOX* family genes

**Fig. 10:**
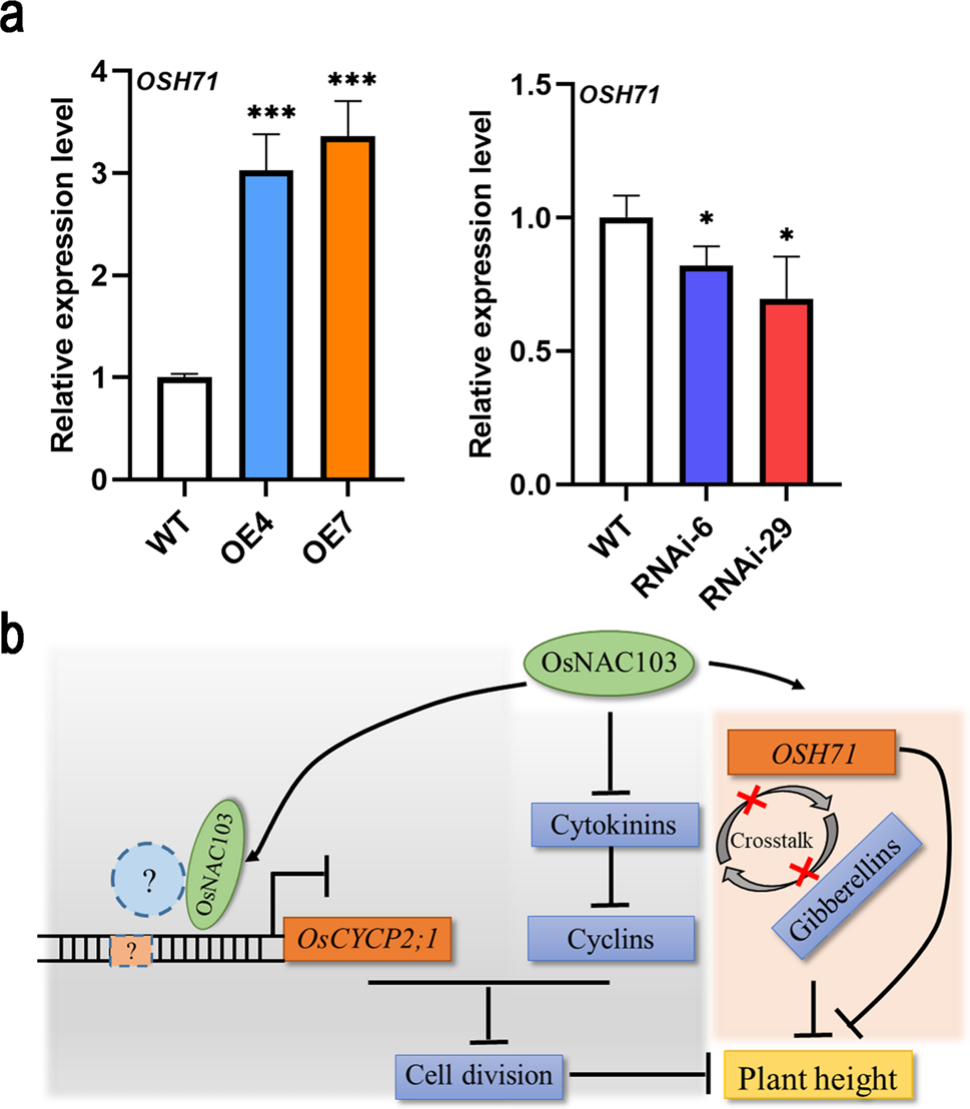
*OsNAC103* is involved in the homeostasis regulation of plant hormones. **a** The relative expression level of *OSH71* in WT, RNAi, and *OE-OsNAC103* plants. Mean values ± SD, *n* = 3. **b** Working model for *OsNAC103* regulating plant height. The circles and rectangles marked with question marks represent unknown proteins and promoter elements, respectively. The WT was used as a control for difference significance analysis. *P < 0.05; **P < 0.01; ***P < 0.001; *t* test

The above findings indicated that both genes associated with cytokinins and gibberellins were impacted. We speculated that *OsNAC103* had disrupted the crosstalk and balance of phytohormones. Therefore, *KNOX* genes involved in gibberellin and cytokinin crosstalk were examined. The results showed that *OSH71* exhibited a significant increase in overexpression plants but was downregulated in RNAi lines, indicating that the balance was regulated by the expression of *OsNAC103* (Fig. 10a). As *OSH71* also plays a significant role in plant morphogenesis, *OsNAC103* may also affect plant growth by regulating *OSH71*. However, OsNAC103 did not directly regulate the expression of *OSH71* (Fig. S5)*.*

## Discussion

The NAC transcription factor family is engaged in diverse processes of plant growth and development. *OsNAC103* overexpression reduced plant height, but there was no significant variation observed in terms of plant height among the WT, RNAi, and *osnac103* mutants. These indicated that *OsNAC103* negatively regulates rice plant height.

NAC family genes can affect the regulation of various phytohormones (Fujita et al. 2004; Bu et al. 2008; Puranik et al. 2012; Mao et al. 2020). Cytokinins play an important role in regulating cell proliferation by positively influencing cell division (Schaller et al. 2014). The reduction in cytokinin levels also inhibits growth (Duan et al. 2019). CKX is the only enzyme that irreversibly degrades cytokinins and nucleosides. In *Arabidopsis thaliana*, the enzymatic activity of CKX4 is enhanced in *35S::AtCKX4* transgenic plants, leading to a reduction in cytokinin levels (Werner et al. 2003). *OsCKX4*-overexpressing plants showed poor agronomic traits, such as dwarfing and late flowering (Gao et al. 2014; Chen et al. 2019), similar to the phenotype of *OE-OsNAC103* plants. The activation of *CKXs* led to a reduction in iP content, while the levels of other active cytokinins remained relatively unchanged compared to those in the WT. The upregulation of *OsARR6* and the repression of *OsBRR1* in plants overexpressing *OsNAC103* inhibited cytokinin signaling. Although the expression of *OsIPTs* increased and that of *OsCKXs* decreased in the RNAi lines, *OsBRR1* was also downregulated, impairing the cytokinin response. This may be one of the reasons why there was no significant difference in plant height between the RNAi lines and WT.

Cytokinins regulate the G1/S and G2/M transitions (Schaller et al. 2014). The decreased expression level of *OsCYCB2.2* also indicated that G2/M was indeed affected. These data indicated that *OsNAC103* negatively regulates cytokinin synthesis, weakens the cytokinin response, and suppresses the expression of genes related to the cell cycle, ultimately leading to a dwarfing phenotype. However, the expression level of *OsCYCP2;1* in *OE-OsNAC103* plants was inconsistent with the transactivation activities of OsNAC103 in rice protoplasts. Similar expression patterns have been reported for OsNAC2 (Chen et al. 2015; Mao et al. 2018) and OsNAC24 (Jin et al. 2023) negatively regulating downstream genes. OsNAC2 may not be the only factor that regulates *OsKO2* and *OsCOX11* expression. Studies have shown that interactions between transcription factors and cofactors may alter DNA-binding affinity and convert activators into repressors (Ren et al. 2021; Li et al. 2023). Based on the previous studies, we deduced that OsNAC103 may cooperate with other proteins to regulate *OsCYCP2;1* expression in plant cells via a more complex mechanism. Furthermore, OsNAC103 may regulate gene expression in a non-linear way with enhancers or silencers, and cofactors on the promoter sequence of *OsCYCP2;1*. The relationships between *OsNAC103*, *OsCKXs* or other proteins, and *OsCYCP2;1* warrant further exploration. In addition, AtCYCP1;1 can interact with AtCDKA1 (Torres Acosta et al. 2004), and OsCYCP4 competes with other typical OsCYCs to bind OsCDKs under phosphate starvation (Xu et al. 2020). The interaction between OsCYCP2;1 and OsCDKs, and the effect of OsNAC103 on protein interaction need further experimental investigations.

Genes associated with gibberellin synthesis were affected in *OsNAC103* overexpression and RNAi plants (Fig. 6a). Changes in intermediate levels of the gibberellin-synthesis pathway are unknown. However, the content of GA_3_, one of the final active gibberellin components, did not vary significantly compared to that in the WT (Fig. S3). Studies have shown that changes in intermediates can also affect the gene expression level regulated by them (Su et al. 2021). In addition, gibberellins also affect the cell cycle (Nagai et al. 2020). In the NAC transcription factor family, *ANAC019* and *ANAC055* participate in the crosstalk between abscisic acid and methyl jasmonate in plant defense response (Bu et al. 2008; Jiang et al. 2009). *OsNAC016* plays a role in maintaining the balance between abscisic acid and brassinosteroids (Wu et al. 2022). Therefore, we believe that OsNAC103 also affects communication between cytokinins and gibberellins.

It can be confirmed that the levels of cytokinins and gibberellin-related genes and *OSH71,* involved in plant hormone regulation, were altered in the *OsNAC103* transgenic lines. The phenotypes of plants overexpressing *KNOX* class I genes were similar to those of plants with excessive cytokinin content (Ori et al. 1999). *OSH71* expression was upregulated in *OE-OsNAC103* plants (Fig. 10a), while the cytokinin content was decreased (Fig. 7a). This suggests that gibberellin–cytokinin crosstalk was affected. In rice, *OSH71* is expressed at a low level in mature and young leaves, but at a relatively high level during panicle and seed development. Ectopic overexpression of *OSH71* causes abnormal plant development and affects the communication between cytokinins and gibberellins. *OsNAC103* may also regulate plant growth by modulating *OSH71* expression. However, the specific regulation mode needs further exploration.

Members of the NAC gene family have functional redundancy. *osnac20–osnac26* double mutants showed decreased storage proteins in the grain, while the single-gene mutant showed no phenotype (Wang et al. 2020). *OsNAC20* and *OsNAC26* co-regulate the synthesis of starch in grains. The RNAi lines and mutant of *OsNAC103* were comparable to WT plants in terms of plant height. The lower transcripts’ accumulation of *OsNAC103* is sufficient to meet normal requirements. On the contrary, the overexpression of *OsNAC103* may have a strong effect on plant development. *OsNAC58* is the homologous gene of *OsNAC103* in rice. *OsNAC58* overexpression plants showed the phenotype of leaf senescence after entering the tillering stage (Liang et al. 2014). Overexpression of *OsNAC103* also promoted leaf yellowing under dark conditions. These suggest a functional similarity between *OsNAC103* and *OsNAC58*. The transgenic plants that OsNAC103 fused with a chimeric dominant repressor (OsNAC103-SRDX) are necessary to generate to explore the functional redundancy problem.

Based on these findings, we propose a working model of *OsNAC103* that regulating plant height in rice (Fig. 10b). When *OsNAC103* was overexpressed, the expression of cytokinin synthetase *IPT* genes was downregulated, and the upregulation of *CKX* genes enhanced the degradation of cytokinins, eventually leading to a reduction in cytokinin content. Upregulated expression of the response factor *OsARR6* inhibits signal transduction. These changes also suppressed the expression of cyclins. In addition, OsNAC103 regulates gene expression by influencing the promoter activity of *OsCYCP2;1*. On the other hand, overexpression of *OSH71* affected the homeostasis of cytokinins and gibberellins and the normal growth of plants. These eventually lead to a dwarfing phenotype in plants. Further research is required to enhance the model.

### Supplementary Information

Below is the link to the electronic supplementary material.

**Supplementary file 1. Fig. S1**: RNA-seq results of the MBKBASE database to analyze the tissue expression pattern of *OsNAC103*. **Fig. S2**: Phenotypes of *osnac103* mutant. **a** Schematic diagram indicating the gRNA location in the genomic region of *OsNAC103 *and the comparison of *osnac103-c2* and *osnac103-c6.* OsNAC103: normal protein sequence. OsNAC103 insertion T: partial protein sequences of mutants inserted with T. OsNAC103 deletion CG: partial protein sequence of a mutant lacking CG. **b** The phenotype of 21-day-old WT (ZH11), *osnac103-c2,* and* osnac103-c6 *plants. Bar = 5 cm. **c** The plant height of 21-day-old WT (ZH11), *osnac103-c2,* and* osnac103-c6 *plants. Mean values ± SD, *n *= 9. **d** The phenotype of WT (ZH11) at the mature stage. Bar = 5 cm. **e** The phenotype of *osnac103-c6* at the mature stage. Bar = 5 cm. **f** The plant height of mature WT (ZH11) and *osnac103-c6 *plants*.* Mean ± SD, *n* = 8. **g** The different internodes of mature WT (ZH11) and* osnac103-c6 *plants (from the top of the stem to the bottom). Bar = 5 cm. **h** The internode lengths of WT (ZH11) and* osnac103-c6 *plants (from the top of the stem to the bottom). Mean ± SD, *n *= 5. ns, no significant difference, *t* test. **Fig. S3**: Analysis of endogenous plant hormone contents in WT and OE plants. Mean values ± SD, *n* = 3. IAA (indole-3-acetic acid), ABA (*cis*-abscisic acid), CS (castasterone), JA (jasmonic acid), JA-ILE (jasmonic acid-isoleucine), *cis*-OPDA (*cis*–12-oxophytodienoic acid), SA (salicylic acid), ACC (1-aminocyclopropane-1-carboxylic acid). **Fig. S4**: Phenotypes of *osnac103* treated with iP and 6-BA. **a** The phenotype and relative shoot length of WT, *osnac103-c2,* and* osnac103-c6 *plants incubated in 1/2 MS medium or treated with different concentrations of iP for 10 days. Bar = 5 cm. Mean values ± SD, *n *= 8. **b** The phenotype and relative shoot length of WT, *osnac103-c2,* and* osnac103-c6 *plants incubated in 1/2 MS medium or treated with different concentrations of 6-BA for 10 days. Bar = 5 cm. Mean ± SD, *n* = 8. The WT was used as a control for significance difference analysis.*P < 0.05; **P < 0.01; ***P < 0.001; ns, no significant difference, *t* test. **Fig. S5**: The regulation between OsNAC103 and *OSH71* promoter was tested by yeast one-hybrid assay. Co-transformant with pB42AD-HY5 and proCOP1 was used as a positive control. **Table S1** Primer sequences were used in the experiment. (PDF 733 KB)

## Data Availability

Data are contained within the article or supplementary material.

## Abbreviations

6-BA: N^6^-benzyladenine
CDK: Cyclin-dependent kinase
CKX: Cytokinin oxidase/dehydrogenase
iP: N^6^-Δ^2^-isopentenyladenine
IPT: Isopentenyl transferase
PAC: Paclobutrazol

## Declarations

### Accession numbers

The RGAP database contains the information on the sequence data. The accession numbers: *OsNAC103* (LOC_Os07g48450), *OsKS* (LOC_Os04g52230), *OsKO2* (LOC_Os06g37364), *OsKAO* (LOC_Os06g02019), *OsCYCB2.2* (LOC_Os06g51110), *OsCYCP1;1* (LOC_Os05g33040), *OsCYCP2;1* (LOC_Os04g53680), *OsIPT3* (LOC_Os05g24660), *OsIPT8* (LOC_Os01g49390), *OsCKX4* (LOC_Os01g71310), *OsCKX5* (LOC_Os01g56810), *OsARR6* (LOC_Os04g57720), *OsBRR1* (LOC_Os03g12350), *OSH71* (LOC_Os05g03884), *ACTIN1* (LOC_Os03g50885).
