# Supplementary material for: OsNAC103, an NAC transcription factor negatively regulates plant height in rice
Source: Planta. 2024 Jan 9;259(2):35. doi: 10.1007/s00425-023-04309-7 (PMC10776745; doi:10.1007/s00425-023-04309-7)
Supplement: Supplementary file 1 — Fig. S1: RNA-seq results of the MBKBASE database to analyze the tissue expression pattern of OsNAC103. Fig. S2: Phenotypes of osnac103 mutant. a Schematic diagram indicating the gRNA location in the genomic region of OsNAC103 and the comparison of osnac103-c2 and osnac103-c6. OsNAC103: normal protein sequence. OsNAC103 insertion T: partial protein sequences of mutants inserted with T. OsNAC103 deletion CG: partial protein sequence of a mutant lacking CG. b The phenotype of 21-day-old WT (ZH11), osnac103-c2, and osnac103-c6 plants. Bar = 5 cm. c The plant height of 21-day-old WT (ZH11), osnac103-c2, and osnac103-c6 plants. Mean values ± SD, n = 9. d The phenotype of WT (ZH11) at the mature stage. Bar = 5 cm. e The phenotype of osnac103-c6 at the mature stage. Bar = 5 cm. f The plant height of mature WT (ZH11) and osnac103-c6 plants. Mean ± SD, n = 8. g The different internodes of mature WT (ZH11) and osnac103-c6 plants (from the top of the stem to the bottom). Bar = 5 cm. h The internode lengths of WT (ZH11) and osnac103-c6 plants (from the top of the stem to the bottom). Mean ± SD, n = 5. ns, no significant difference, t test. Fig. S3: Analysis of endogenous plant hormone contents in WT and OE plants. Mean values ± SD, n = 3. IAA (indole-3-acetic acid), ABA (cis-abscisic acid), CS (castasterone), JA (jasmonic acid), JA-ILE (jasmonic acid-isoleucine), cis-OPDA (cis–12-oxophytodienoic acid), SA (salicylic acid), ACC (1-aminocyclopropane-1-carboxylic acid). Fig. S4: Phenotypes of osnac103 treated with iP and 6-BA. a The phenotype and relative shoot length of WT, osnac103-c2, and osnac103-c6 plants incubated in 1/2 MS medium or treated with different concentrations of iP for 10 days. Bar = 5 cm. Mean values ± SD, n = 8. b The phenotype and relative shoot length of WT, osnac103-c2, and osnac103-c6 plants incubated in 1/2 MS medium or treated with different concentrations of 6-BA for 10 days. Bar = 5 cm. Mean ± SD, n = 8. The WT was used as a control [file 425_2023_4309_MOESM1_ESM.pdf]

Supplementary data

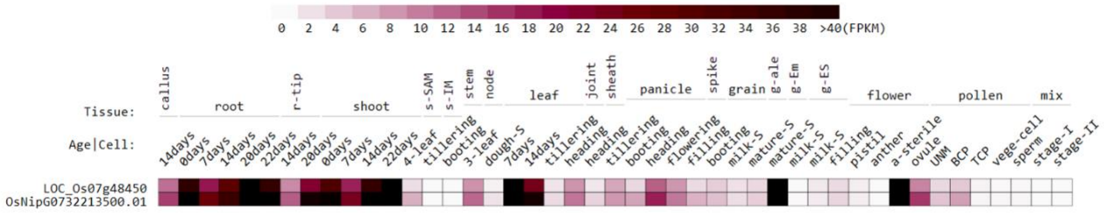

Fig. S1 RNA-seq results of the MBKBASE database to analyze the tissue expression pattern of *OsNAC103*.

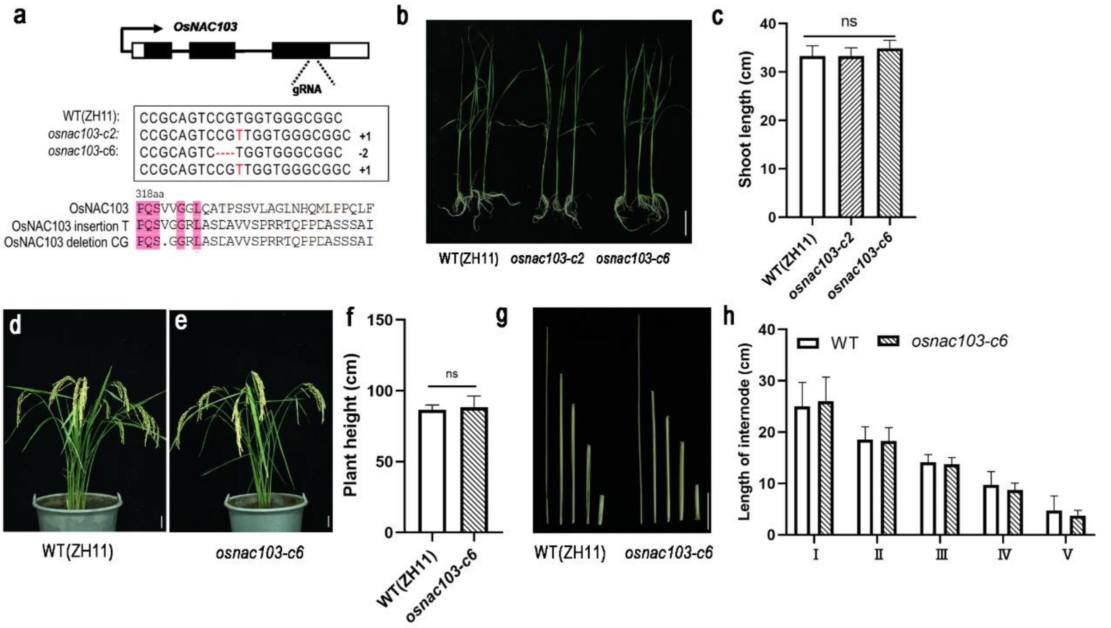

Fig. S2 Phenotypes of *osnac103* mutant. **a** Schematic diagram indicating the gRNA location in the genomic region of *OsNAC103* and the comparison of *osnac103-c2* and *osnac103-c6.* OsNAC103: normal protein sequence. OsNAC103 insertion T: partial protein sequences of mutants inserted with T. OsNAC103 deletion CG: partial protein sequence of a mutant lacking CG. **b** The phenotype of 21-day-old WT (ZH11), *osnac103-c2,* and *osnac103-c6* plants.

Bar = 5 cm. **c** The plant height of 21-day-old WT (ZH11), *osnac103-c2,* and *osnac103-c6* plants. Mean ± SD, *n* = 9. **d** The phenotype of WT (ZH11) at the mature stage. Bar = 5 cm. **e** The phenotype of *osnac103-c6* at the mature stage. Bar = 5 cm. **f** The plant height of mature WT (ZH11) and *osnac103-c6* plants. Mean ± SD, *n* = 8. **g** The different internodes of mature WT (ZH11) and *osnac103-c6* plants (from the top of the stem to the bottom). Bar = 5 cm. **h** The internode lengths of WT (ZH11) and *osnac103-c6* plants (from the top of the stem to the bottom). Mean ± SD, *n* = 5. ns, no significant difference, t-test.

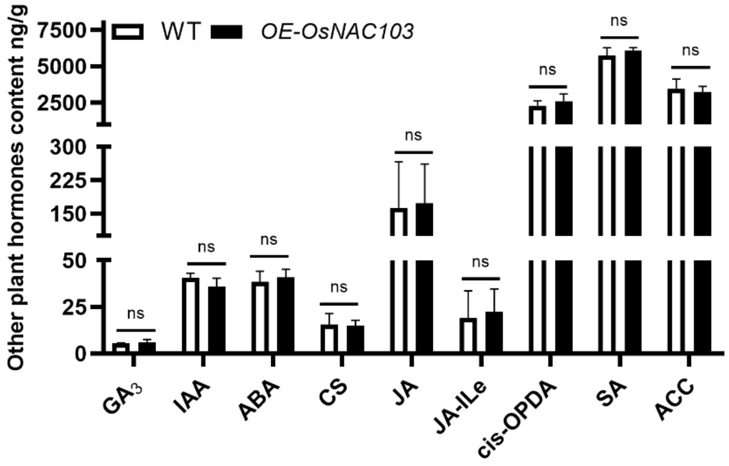

Fig. S3 Analysis of endogenous plant hormone contents in WT and OE plants. Mean ± SD, n = 3. IAA (indole-3-acetic acid), ABA (*cis*-abscisic acid), CS (castasterone), JA (jasmonic acid), JA-ILE (jasmonic acid-isoleucine), *cis*-OPDA (*cis*–12-oxophytodienoic acid), SA (salicylic acid), ACC (1-aminocyclopropane-1-carboxylic acid).

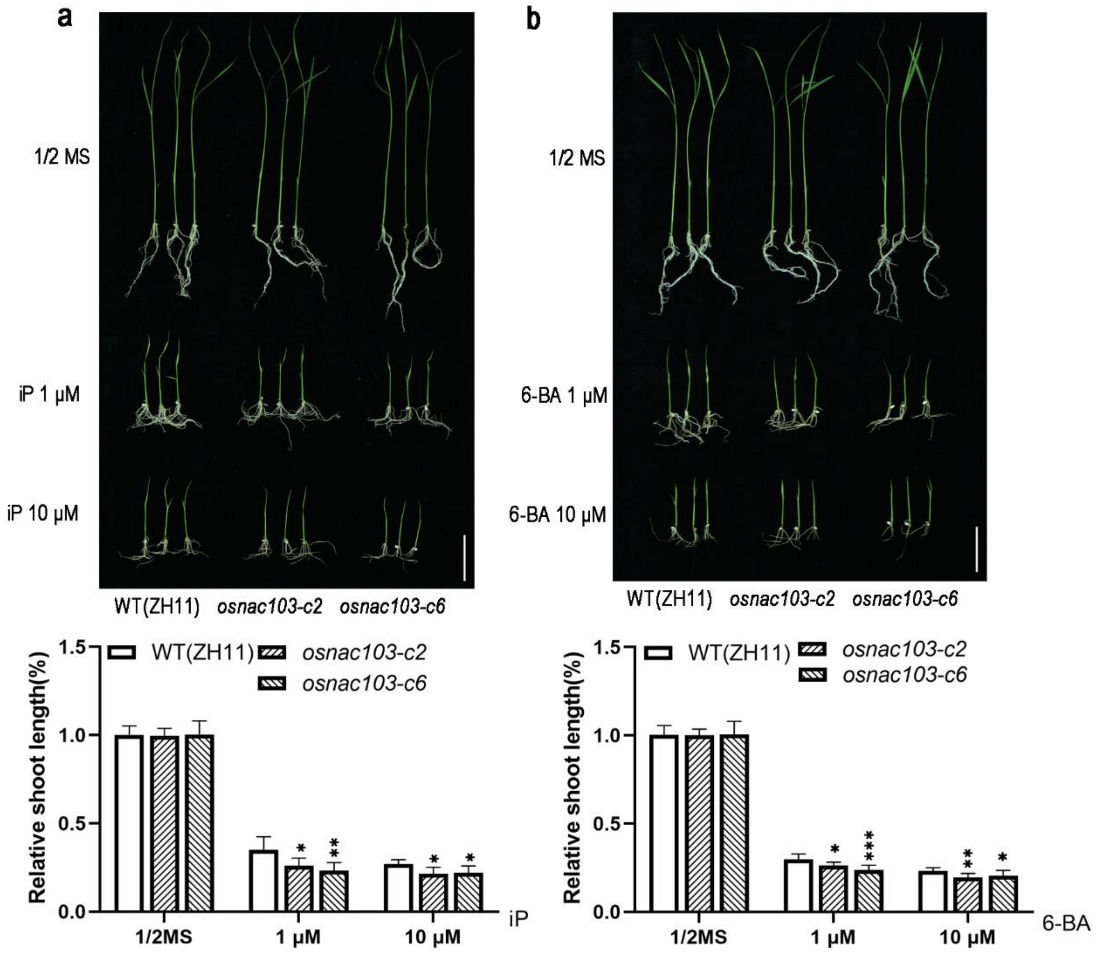

Fig. S4 Phenotypes of *osnac103* treated with iP and 6-BA. **a** The phenotype and relative shoot length of WT, *osnac103-c2,* and *osnac103-c6* plants incubated in 1/2 MS medium or treated with different concentrations of iP for 10 days. Bar = 5 cm. Mean ± SD, $n = 8$. **b** The phenotype and relative shoot length of WT, *osnac103-c2,* and *osnac103-c6* plants incubated in 1/2 MS medium or treated with different concentrations of 6-BA for 10 days. Bar = 5 cm. Mean ± SD, $n = 8$. The WT was used as a control for significance difference analysis.*, $P < 0.05$; **, $P < 0.01$; ***, $P < 0.001$; ns, no significant difference, t-test.

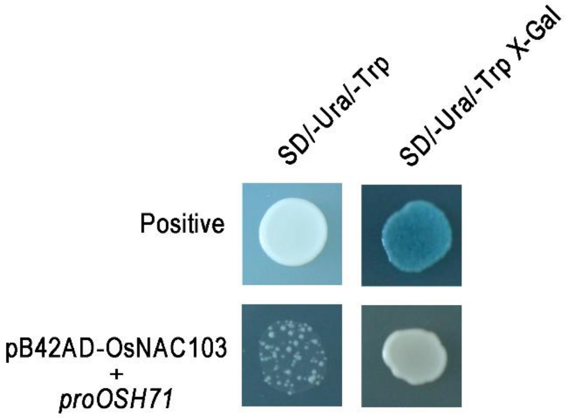

Fig. S5 The regulation between OsNAC103 and *OSH71* promoter was tested by yeast one-hybrid assay. Co-transformant with pB42AD-HY5 and proCOP1 was used as a positive control.

Table S1. Primers used in this work.	
**Primers used for qRT-PCR**	
OsKS-F	TGTGCTCCCGACTCTGTATTTC
OsKS-R	TGTCTATGGATTTCTGGGCTTTC
OsKAO-F	CTGCGTCAACATCTCCTTCGT
OsKAO-R	CACAGTCTTGCTCCCAGTCCA
OsKO2-F	TGAAGTAGCCAAGGAGGCGA
OsKO2-R	CGCTGATTGCGACCATACTTT
OsCYCP1;1-F	AACAAGTTCACGGCGTCAATAG
OsCYCP1;1-R	GGATGTAAAATCAAGTTGGTCAGC
OsCYCP2;1-F	AAGAGCGCGTTCTGTGTGTA
OsCYCP2;1-R	TGCTGAACAGGCAACGAGTA
OsCYCB2.2-F	CTCAAGGCTGCACAATCTGACA
OsCYCB2.2-R	GCATTGACGGCTGGAATTTG
OsIPT3-F	GAGCTGTGCTTCCTGTGGGTGGACT
OsIPT3-R	GCGACCTTGTACTTGTCTCCGTGCG
OsIPT8-F	CGGGGAAGACCAAGCTTT
OsIPT8-R	GAGCGACACCTTGTTCGT
OsCKX4-F	TGGTGCGGTGGATACGTGTTCT
OsCKX4-R	TGGGTCCTGTGGCTTGAACG
OsCKX5-F	TTGTGACAGGGAGGGGAGAA
OsCKX5-R	GAGAACACGTATCCACCGCA
OsARR6-F	GTGGTGATCATGTCGTCGGA
OsARR6-R	ATCTGATACGGCTGCAGAGC
OsBRR1-F	CTAATCCATTCTGCCCGCCT
OsBRR1-R	GGTCCCTTCCAGGCAATACC
OsNAC103-F	GGCCCTCGTCTTCTACCGCG

OsNAC103-R	CCCAGTCGTCCAGCCTCATGG
ACTIN1-F	CCAGCAAGGTCGAGACGAA
ACTIN1-R	TGTATGCCAGTGGTCGTACCA
OSH71-F	TTCATGGAGACGTACTGCCG
OSH71-R	CGGCTCATCCTCTGAAGACC
**Primers used for vector construction(Underscores identify sequences used for vector connections)**	
proOsNAC103-GUS-F	TAAATTCAATATCTTCCCAACCGTA
proOsNAC103-GUS-R	CGACGATCTAATACGGTTACT
proOsCYCP2;1-LUC-F	TCGACGGTATCGATAAGCTTGCAAATGCAACTGATTCTC
proOsCYCP2;1-LUC-R	GCTCTAGAACTAGTGGATCCGGCCATGCACGGCATCGA
OsNAC103-CDS-F	ATGGAGATGACGATGTCGTCGG
OsNAC103-CDS-R	TCAGAATAGCTGAGGAGGAAG
proOSH71-placzi-F	TTGATATTGGATCGGAATTCTTTCAAATTAACATTTCCTGG
proOSH71-placzi-R	CGGGTACCGAGCTCGAATTCGTCCAATCCCCTCTTCCCCCA
OsNAC103-PB42AD-F	ATTATGCCTCTCCCGAATTCATGGAGATGACGATGTCGTCG
OsNAC103-PB42AD-R	TTCTCGAGTCGGCCGAATTCTCAGAATAGCTGAGGAGGAAGC
BD-OsNAC103$^{\triangle C}$-R	CGACGGATCCCCGGGAATTCGAGGCGGTACTCGTGCATGA
BD-OsNAC103$^{\triangle N}$-F	TGGCCATGGAGGCCGAATTCGCCGCCGACCCTCTCGCCGC
BD-OsNAC103-C$^{\triangle C1}$-R	CGACGGATCCCCGGGAATTCCGGGTAGCACTCCGCGAGG
BD-OsNAC103-C$^{\triangle C2}$-R	CGACGGATCCCCGGGAATTCGTAGAAGCTGTAGGCGTCGTC
BD-OsNAC103-C$^{\triangle C3}$-R	CGACGGATCCCCGGGAATTCCATCATCGGCGACGCCTG
BD-OsNAC103-C$^{\triangle C4}$-R	CGACGGATCCCCGGGAATTCGACGTTGCGGAATCGGGAG
BD-OsNAC103-F	TGGCCATGGAGGCCGAATTCATGGAGATGACGATGTCGTC
BD-OsNAC103-R	CGACGGATCCCCGGGAATTCTCAGAATAGCTGAGGAGGAA
**Primers used for EMSA**	
OsCYCP2;1-P1-F-Texas Red	AAACCATTTCGGATCACGATTTTGGCACGGAATGCCGACAAAAT

OsCYCP2;1-P2-F-Texas Red	CTTCATCGGACGGCCACGGCCCCACCGCCGCCGCATCATCCAC
OsCYCP2;1-P3-F-Texas Red	AGCTAACGCGCGCCACTCCACGCTGTCGATGCCGTGCATGGCC
OsCYCP2;1-P1-R	ATTTTGTCGGCATTCCGTGCCAAAATCGTGATCCGAAATGGTTT
OsCYCP2;1-P2-R	GTGGATGATGCGGCGGCGGTGGGGCCGTGGCCGTCCGATGAAG
OsCYCP2;1-P3-R	GGCCATGCACGGCATCGACAGCGTGGAGTGGCGCGCGTTAGCT
